# Bactericidal and anti-inflammatory effects of *Moquilea tomentosa* Benth. flavonoid-rich leaf extract

**DOI:** 10.1186/s12906-023-03968-z

**Published:** 2023-05-10

**Authors:** Mariana Freire Campos, Leopoldo Clemente Baratto, Vinícius Mendes Vidal, Ivana Ventura Nascimento, Brendo Araujo Gomes, Genes de Lima Martins Neto, Priscilla Christina Olsen, Rodrigo Ribeiro Tarjano Leo, Lilian Oliveira Moreira

**Affiliations:** 1 Laboratório de Anatomia Vegetal, Departamento de Botânica, Federal University of the State of Rio de Janeiro (UNRIO), Rio de Janeiro, Brazil; 2 Laboratório de Farmacognosia Aplicada, Departamento de Produtos Naturais e Alimentos, Federal University of Rio de Janeiro (UFRJ), Rio de Janeiro, Brazil; 3 Laboratório de Bacteriologia e Imunologia Clínica (LABIC), Departamento de Análises Clínicas e Toxicológicas, Faculdade de Farmácia, Federal University of Rio de Janeiro (UFRJ), Rio de Janeiro, Brazil; 4 Laboratório de Estudos em Imunologia (LEI), Departamento de Análises Clínicas e Toxicológicas, Faculdade de Farmácia, Federal University of Rio de Janeiro (UFRJ), Rio de Janeiro, Brazil; 5 Laboratório de Bacteriologia e Imunologia Clínica (LABIC), Departamento de Análises Clínicas e Toxicológicas, Faculdade de Farmácia, Federal University of Rio de JaneiroRua Professor Paulo Rocco, Bloco A2-07, Centro de Ciências da Saúde (CCS), UFRJ, Cidade Universitária, Rio de Janeiro, RJ CEP: 21941-902 Brazil

**Keywords:** Flavonoid-*O*-glycosides, Mass spectrometry, Antibacterial activity, Natural products, Biofilm, Cytokines

## Abstract

**Background:**

Natural products are an important source of bioproducts with pharmacological properties. Here we investigate the components of leaves from *M. tomentosa* Benth. (Fritsch) (Chrysobalanaceae) and its effects on bacterial cell growth, biofilm production and macrophage activity.

**Methods:**

The effect of the different leaf extracts against bacterial cell growth was performed using the microdilution method. The most active extract was analyzed by mass spectrometry, and its effect on bacterial biofilm production was evaluated on polystyrene plates. The extract effect on macrophage activity was tested in the RAW264.7 cell line, which was stimulated with different concentrations of the extract in the presence or absence of LPS.

**Results:**

We show that the ethyl acetate (EtOAc) extract was the most effective against bacterial cell growth. EtOAc extract DI-ESI (-)MS^n^ analysis showed the presence of a glycosylated flavonoid tentatively assigned as myricetin 3-*O*-xylosyl-rhamnoside (MW 596). Also, the EtOAc extract increased biofilm formation by *S. aureus* and inhibited cytokine and NO production induced by LPS in RAW macrophages.

**Conclusion:**

*M. tomentosa* flavonoid-enriched EtOAc extract presented a bactericidal and anti-inflammatory pharmacological potential.

**Supplementary Information:**

The online version contains supplementary material available at 10.1186/s12906-023-03968-z.

## Background

Native Brazilian flora is an important source of bioactive natural products, including antimicrobial and anti-inflammatory substances [43]. According to [29], about 65% of the new drugs discovered between 1980 and 2014 were secondary metabolites of plants.

Most of the studies regarding the antibacterial activity of natural products focus on bacterial death or growth inhibition. However some of these products are able to inhibit bacterial virulence factors, such as biofilms [33]. Biofilms are a sessile community of bacteria within a polymeric extracellular matrix secreted by bacteria. These structures may help bacterial colonization and survival, protecting the microorganisms against antimicrobials and the immune system [18, 33].

Medicinal plants are commonly used as anti-inflammatory alternative treatments, mainly in Asia. Several in vitro and in vivo studies have confirmed the ability of immune modulation by purified molecules from natural origin (Revised by [13]). This is of particular interest since several commercial anti-inflammatory drugs have deleterious side effects, highlighting the importance of new drug discovery.

Approximately 167 products of secondary plant metabolism have been described in species of the Chrysobalanaceae family. Their secondary metabolism products are mainly composed of terpenoids and flavonoids [8, 16].Studies have shown the traditional use of some Chrysobalanaceae species in Brazil and worldwide. Species belonging to *Licania*, *Microdesmia* and *Moquilea* genus are the most studied and presented different biological activities and uses in popular medicine [16].

*Moquilea tomentosa* Benth. is a Brazilian tree species, popularly known as "oiti" or "oitizeiro". It naturally grows in the Northeast region of the country; however it is widely used as a shading tree for afforestation due to its relatively low trunk and globular crown being common in several urban areas in Brazil [2, 9]. In traditional medicine, *M. tomentosa* is used for intestinal and stomach disorders [38].

Fewer studies have shown that *M. tomentosa* extracts present different biological activities in vitro, such anti-herpetic [27], antioxidant [15, 25, 31, 38], antibacterial [15, 38], anti-mite [42] and cytotoxicity against tumor cells [17]. Since *M. tomentosa* is used for intestinal and stomach disorders in traditional medicine it was suggested that this effect could be related to the plant antibacterial effect observed in vitro [38]. However, it remains unknown if the pharmacological effect in intestinal disorders could be also related to an anti-inflammatory effect of the plant extract.

Although previous chemical analysis of leaves and fruits from *M. tomentosa* have been performed [9], little is known about the molecular characteristics of their extracts with biological activity. Here we investigated the in vitro biological effects of crude extract and different fractions of *M. tomentosa* leaves on bacterial survival, bacterial biofilm production and macrophage activity. Moreover, we investigated the chemical profile of the most active fraction by DI-ESI (-) Ms^n^.

## Methods

### Plant material collection and extraction

Leaves of three individuals of *M. tomentosa* were collected at the Praia Vermelha campus of the Federal University of the State of Rio Janeiro (UNIRIO). A voucher specimen was deposited in the UNIRIO Herbarium – Prof. Jorge Pedro Pereira Carauta (HUNI), under the number HUNI3770. Leaves were dried at 45ºC and then ground. The pulverized material was extracted by static maceration at room temperature (RT) using 96° GL ethanol, stirring periodically. The crude ethanolic extract (EtOH) was concentrated under reduced pressure on a rotary evaporator. Subsequently, 5 g of crude extract was solubilized in 100 mL of methanol: water (9:1) solution and then partitioned by liquid–liquid extraction using (n-hexane, dichloromethane (CH_2_Cl_2_), ethyl acetate (EtOAc) and n-butanol (BuOH)). Stock solutions were prepared by suspending 50 mg of crude extract and the fractions in 1 mL of sterile 100% dimethyl sulphoxide (DMSO).

### Antibacterial activity

To investigate the antibacterial activity of the extracts the broth microdilution method was used, following NCCLS-M07-A9 recommendations [12]. Twelve bacterial species were tested: four *Staphylococcus aureus* strains, ATCC 29213, ATCC 12600, ATCC 25923, ATCC 33591; *Staphylococcus epidermidis* ATCC 12228; *Staphylococcus simulans* ATCC 27851, *Streptococcus mutans* ATCC 26285, *Escherichia coli* ATCC 35218, *Shigella sonnei* ATCC 1484, *Klebsiella pneumoniae* ATCC 700603, *Proteus hauseri* ATCC 13315; *Pseudomonas aeruginosa* ATCC 27853 (Table 1). Bacteria were cultured in Trypticase Soy Agar (TSA) for 18 h at 37ºC, and the inoculum was prepared in phosphate buffered saline (PBS) pH 7.2 at OD_600 nm_ = 0.1. Five microliters of the inoculum were distributed in 96-well polystyrene microplates, and mixed with the diluted extracts with concentrations varying from 7.8 to 1000 μg/mL, with a final volume of 100μL. The reference antimicrobials vancomycin and gentamycin were used as positive controls (data not shown) [12]. Microplates were then incubated for 24 h at 37 °C, and the Minimum Inhibitory Concentration (MIC) determined as the lowest concentration where it was not possible to detect bacterial growth visually (turbidity). The Minimum Bactericidal Concentration (MBC) was determined by adding 20 μL of 0.01% resazurin (Sigma) per well, followed by a 2 h incubation period at 37ºC. The MBC was defined as the lowest concentration in which there was reduction of the resazurin added to the system [14].

**Table 1:** Minimum Inhibitory Concentration (MIC) and Minimum Bactericidal Concentration (MBC) values of *L. tomentosa* leaf extracts (μg/mL) against 12 bacterial strains

**Samples**^**a**^** / ATCC number**	**Partition**									
	**Hexane**		**CH**_**2**_**Cl**_**2**_		**EtOAc**		**Butanol**		**Ethanol**	
	**MIC**	**MBC**	**MIC**	**MBC**	**MIC**	**MBC**	**MIC**	**MBC**	**MIC**	**MBC**
**Gram-positive**										
*Staphylococcus aureus*/ 29213	> 1000	> 1000	> 1000	> 1000	> 1000	> 1000	> 1000	> 1000	> 1000	> 1000
*Staphylococcus aureus /* 12600	1000	> 1000	1000	> 1000	125	250	> 1000	> 1000	500	> 1000
*Staphylococcus aureus /* 33591	> 1000	> 1000	> 1000	> 1000	250	250	> 1000	> 1000	500	> 1000
*Staphylococcus aureus /* 25923	> 1000	> 1000	> 1000	> 1000	> 1000	> 1000	> 1000	> 1000	> 1000	> 1000
*Staphylococcus epidermidis/* 12228	> 1000	> 1000	> 1000	1000	250	250	1000	1000	> 1000	> 1000
*Staphylococcus simulans /* 27851	> 1000	> 1000	> 1000	> 1000	250	250	> 1000	> 1000	500	> 1000
*Streptococcus mutans /* 26285	> 1000	> 1000	> 1000	> 1000	125	125	> 1000	> 1000	500	> 1000
**Gram-negative**										
*Escherichia coli /* 35218	> 1000	> 1000	> 1000	> 1000	> 1000	> 1000	> 1000	> 1000	1000	> 1000
*Shigella sonnei /* 1484	1000	> 1000	250	> 1000	> 1000	> 1000	> 1000	> 1000	500	> 1000
*Klebsiella pneumoniae /* 700603	1000	> 1000	1000	> 1000	> 1000	> 1000	1000	> 1000	1000	> 1000
*Proteus hauseri /* 13315	> 1000	> 1000	500	> 1000	125	125	> 1000	> 1000	500	> 1000
*Pseudomonas aeruginosa /* 27853	1000	ND	1000	ND	1000	ND	1000	ND	1000	ND

Values in µg/ mL

*ND* Not determined

^a^The data are representative of an experiment carried out at least three times independently

### Biofilm production

To investigate the extract effect on the bacterial biofilm production, the assay was performed in 96-well flat well polystyrene plates, as previously described [39]. Briefly, 5μL of bacterial suspension at OD_600nm_ = 0.1, were mixed with TSB media containing the dilute extract (7.8 – 1000 μg/mL) and incubated for 18 h at 37ºC. The content was carefully aspirated and the wells were washed twice with PBS solution pH 7.2. Then the plates were heated at 60 °C for 1 h for biofilm fixation, and 150μL of an aqueous solution of 0.1% safranin was added per well, following 15 min incubation at RT. Then, the plate was washed with PBS twice, and 150 μL of DMSO was added per well for 30 min at RT, and read at the spectrophotometer (Multiscan GO, ThermoScientific) at 492 nm. The biofilm producer strains *S. aureus* (ATCC25923 and ATCC29213) were used as positive controls for the assay.

### Macrophage culture, viability and stimuli for NO and cytokine detection

To evaluate the effects of the extract on the modulation of inflammatory response of macrophages, we used the murine macrophage cell line RAW264.7. The cells were cultured in DMEM complete medium containing 10% fetal bovine serum and antibiotics penicillin/streptomycin (Gibco, NY, USA), at 37 °C with 5% CO_2_. Macrophage viability was investigated through the MTT assay.Cells (5 × 10^5^ cells*/*mL) were plated at 96-well plates and treated with different concentrations of the partition (7.8 – 1000 μg/mL), diluted at DMEM at the final volume of 100μL, following an incubation at 37 °C with 5% CO_2_ for 18 h. Then, the supernatants were collected, fresh media was added, and 10 μL MTT (5 mg/mL) added per well, following 4 h incubation at 37 °C. The supernatant was discarded and DMSO was added (150μL*/* well). The plate was read at 540 nm using a spectrophotometer (Multiscan GO, ThermoScientific). For the stimuli, the following conditions were tested: (i) 250 μg/mL EtOAc extract pre-treatment for 1 h, then media removal and LPS (1 μg/mL) was added (Sigma) for 18 h; (ii) 250 μg/mL EtOAc extract plus LPS; (iii) LPS alone; (iv) EtOAc extract alone [19]. The LPS was used as a positive control for nitric oxide (NO) and cytokine production, After 24 h of incubation, the supernatant was collected for NO/nitrite and cytokines (IL-6 and TNF-α) detection, using the Griess reagent and ELISA kit, respectively. ELISAs were performed as instructed by the manufacturer (Invitrogen—Thermo Fisher Scientific).

### Thin layer chromatography (TLC) and chemical analysis by mass spectrometry

The chemical characteristics of the most active fraction was investigated by thin layer chromatography (TLC) and mass spectrometry. TLC was performed in silica gel 60 chromatoplates (5 cm), and the material was eluted with ethyl acetate: acetone: water (25:8:2) or pure ethyl acetate. After, the plates where treated with the chromogenic solution NP-PEG (diphenylboriloxietilamine in methanol 1.0% (NP) + polietilenoglycol 4000 in ethanol 5.0% (PEG), then the plates were heated at 60 °C. For the MS analysis, the EtOAc fraction was diluted in acetonitrile:MeOH (1:1 v/v) in 10μL, and the solvents used were a mixture of water, 0.1% NaOH and methanol. The direct infusion mass spectrometry (DI-ESI-IT-MS) analysis was performed using a LCQ Fleet (ThermoFisher Scientific, Waltham, Massachusetts, USA). The mass spectrometer (MS), equipped with an electrospray source (ESI) and Ion Trap analyzer (with 1000 resolution), was operated in the negative ion mode. The full scan data acquisition (mass range: *m/z* 50–1500) was used at a sample flow rate of 10 µL min^−1^. Substance annotation was performed as recommended based on MS and MS^2^ spectra [40].

### Statistical analysis

All data were expressed as means ± SD. Comparison were made using T- student test or one-way ANOVA with Dunnet post test, and differences were considered significant with p < 0.05. Data were analyzed using the GraphPad Prism 8 (Graphpad Software, San Diego, CA, USA).

## Results

### Antibacterial activity of *M. tomentosa* leaf crude extract and fractions

The effect of the different *M. tomentosa* crude extract and fractions on bacterial survival was investigated, and the MIC and MBC values were determined (Table 1). Overall, the EtOAc fraction showed the highest activity leading to the lowest MIC and MBC values for different bacterial strains (*S. epidermidis* ATCC12228, *S. aureus* ATCC12600, *S. simulans* ATCC 27851, *S. mutans* ATCC 26285). It was observed that Gram-positive bacteria were more susceptible to most active fractions, while the Gram-negative strains were resistant, with the exception of the *P. hauseri* ATCC 13315 (MIC and MBC = 125 µg/mL, EtOAc fraction). The CH_2_Cl_2_ fraction showed activity only for *S. sonnei* ATCC 1484, revealing a strong bacteriostatic action (MIC = 250 µg/mL), while n-hexane and BuOH fractions showed no activity. The crude ethanolic extract presented a bacteriostatic effect, with MICs of 500 μg/mL and MBCs higher than 1000 μg/mL (Table 1).

### Chemical analysis of *M. tomentosa* leaves EtOAc fraction

Since EtOAc fraction of *M. tomentosa* leaves was the most effective on bacterial cell growth, we investigated its chemical profile composition by TLC and mass spectrometry. TLC analysis of EtOAc fraction using NP-PEG with different elution systems, showed the presence of several orange – yellow bands which indicated the presence of flavonoids (Figure S1). The DI-ESI-IT-MS- negative mode full spectrum showed a major compound at *m/z* 595, and other few minor ionic species (Figure S2A). The MS^2^ of ion *m/z* 595 fragmentation generated a major ionic species, *m/z* 316 (Figure S2B), and the MS^3^ analysis of the ion *m/z* 316 generated *m/z* 287, 271 and 270 as major peaks (Figure S2 C). Based on the fragmentation pattern [40] of the ion *m/z* 595 and chemotaxonomic comparisons with other flavonoids identified previously in other Chrysobalanaceae species (Table S1) [3, 4], these results suggest the major compound of the EtOAc fraction of *M. tomentosa* leaves is a glycosylated flavonoid tentatively assigned as myricetin 3-*O*-xylosyl-rhamnoside (MW 596).

### Activity of *M. tomentosa* leaves EtOAc fraction on bacteria biofilm production

**Fig. 1:**
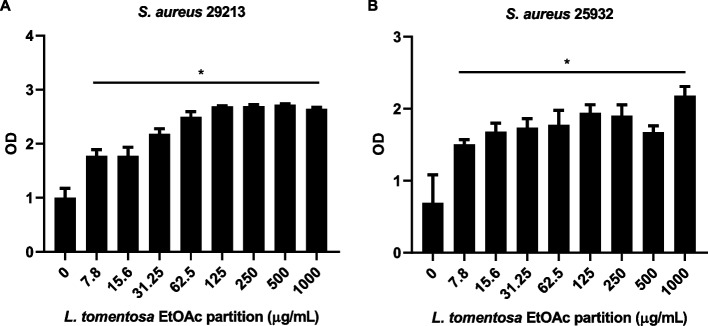
Effect of the ethyl acetate partition of *M. tomentosa* on the formation of S*taphylococcus aureus* biofilm. **A** *S. aureus* ATCC 29,213; **B** *S. aureus* ATCC 25,932. Tested concentrations from 7.8 to 1000 μg /mL. The values shown correspond to averages resulting from independent experiments repeated at least three times. * Significant difference (*p*-value < 0.05) when compared to the control (0)

Although some bacterial strains survived the treatment, we decided to investigate the effects of the EtOAc fraction on bacterial biofilm production, using two biofilm producers *S. aureus* (ATCC25923 and ATCC29213) strains that were resistant to this fraction (Table 1). It was shown that the treatment with different concentrations of the EtOAc fraction significantly increased the biofilm production by both strains (Fig. 1).

### Effect of the EtOAc fraction on macrophage survival and NO and cytokine production

**Fig. 2:**
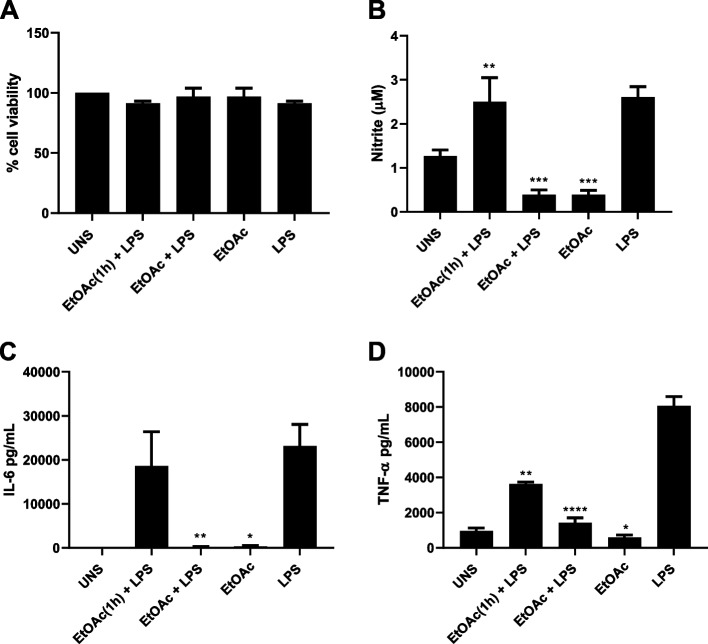
Effect of the ethyl acetate partition of *M. tomentosa* on RAW macrophages. **A** RAW cell viability by MTT; **B** NO, **C** IL-6 and **D** TNF-α production after 24 h incubation with the different conditions; UNS, unstimulated; EtOAc (1 h) + LPS, 250 μg/mL EtOH partition pre-treatment for 1 h, then media removal and LPS (1 μg/mL) added, for 18 h; EtOAc + LPS, 250 μg/mL EtOH partition plus LPS (1 μg/mL), for 18 h; EtOH, partition alone; LPS alone

Previous studies have shown that myricetin compound was able to inhibit the LPS stimulatory response on macrophages [19]. To investigate the ability of the EtOAc fraction of *M. tomentosa* leaves to modulate macrophage activity, RAW cells were stimulated with this fraction, in the presence or absence of LPS. The EtOAc fraction treatment was toxic for RAW cells at 500 and 1000 μg/mL (Fig. 2A), then we decided to use 250 μg/mL to stimulate RAW cells, since it was the highest concentration in which cell death was not detected. Pre-incubation of macrophages with the EtOAc fraction for 1 h and subsequent addition of LPS for 24 h was able to reduce the LPS induced NO (*p* < 0.0028) and TNF-α (*p* < 0.0001), but not IL-6. The addition of the EtOAc fraction with LPS for 24 h reduced the LPS ability to induce NO (*p* < 0.0001), TNF-α (*p* < 0.0001) and IL-6 (*p* < 0.0013) production. The addition of the EtOAc fraction alone, was able to reduce the basal production (unstimulated) of NO (*p* < 0.0001), but not of IL-6 or TNF- α (Fig. 2B, C and D).

## Discussion

In the context of an infection disease, it is important to eliminate the pathogen, and also minimize the immune response induced by the infectious agent, since the immune response can cause tissue damage, in certain circumstances. Thus, both aspects are related, and worthy to be investigated. Since *M. tomentosa* is used for intestinal disorders in traditional medicine, we decided to investigate both the anti-bacterial and anti-inflammatory properties of the plant leaf extract.

A previous study has suggested that the plant effect in intestinal tract could be due to the presence of flavonoids with antibacterial activity in the leaves [38].

Flavonoids are a very diverse class of natural products that present a wide range of biological activities such as antibacterial, antiviral, antioxidant, antitumor, antithrombotic, and immunomodulatory [23]. Flavonoid structure consists in two benzene rings, A and B, plus a heterocyclic pyrane ring that is usually glycosylated in plants [32]. In general, phenolic compounds (such as flavonoids and tannins) and saponins are mostly recovered from polar partitions, such as in EtOAc, when liquid–liquid extraction is used [10]. Several flavonoids have been described for the *Licania* and other Chrysobalanaceae genera, mainly myricetin, quercetin and kaempferol [8, 16].

Previous studies showed that *M. tomentosa* hydroalcoholic leaf extracts were active against different Gram-positive and -negative bacteria, with MIC values ranging from 32 to ≥ 512 μg/mL [38]. Ethanol extraction of *M. tomentosa* seeds also showed bactericidal activity [15]. In both studies, the extracts used were crude and non-partitioned, limiting the systematic investigation of the molecules involved in the bactericidal effect.

Here we observed that the EtOAc partition presented the strongest antibacterial activity, with concentrations varying from 125 to 500 μg/mL (Table 1). Interestingly, the EtOAc partition was more active against Gram-positive than Gram-negative bacteria (Table 1). According to [37], natural products that lead to MIC values lower than 500 μg/mL have strong bioactive and pharmaceutical potentials. Thus, our results confirmed the antibacterial ability of *M. tomentosa*, and showed that this activity was present at the EtOAc fraction. It is important to point that, Gram-positive bacteria are less relevant than Gram-negatives in the context of intestinal disorders, and although the EtOAc partition was less effective against Gram-negative, we tested only few reference strains. For better correlation of traditional medicine use of *M. tomentosa* and its effect in intestinal bacteria, more clinical Gram-negative isolates should be investigated.

The mass spectrometry analysis of the most active *M. tomentosa* EtOAc fraction showed the presence of a major compound (*m/z* 595), tentatively assigned as a flavonol glycoside myricetin 3-*O*-xylosyl-rhamnoside. The annotation of this compound was based on previously putatively annotated compounds with no chemical standard reference, based on spectral similarities following the Metabolomics Standards Initiative (MSI) recommendations [40]. The observation of the ion *m/z* 595 fragmentation revealed the formation of two major ionic species, *m/z* 316 and *m/z* 271, where the ion *m/z* 316 [M-279]^−^ is related to the loss of the disaccharide xylosyl-rhamnoside moiety, corresponding to the aglycone myricetin [20]. The MS^3^ data of ion *m/z* 316 showed fragments *m/z* 271 [M-H–CO-H_2_O]^−^ and *m/z* 179, typically found in retro Diels–Alder reactions of flavan-3-ols with a dihydroxilated-A ring, a characteristic of myricetin derivatives [34].

Myricetin can be isolated from different plant sources such as berries, vegetables and herbs, and it may occur in both free and glycosylated forms. Several myricetin derivatives have been identified in the Chrysobalanaceae family [3-8, 18] (Table S1). Bilia and co-workers also reported a glycosylated myricetin with *m/z* 595 identified as myricetin-3-(2’-xylosyl)-rhamnoside, in both *L. carii* [4] and *Moquilea pyrifolia* [3] (Table S1) species. Whether the *M. tomentosa* myricetin 3-*O*-xylosyl-rhamnoside compound presents similar glycosylation sites remains to be investigated.

Environmental stress conditions such as the presence of certain antibiotics, influences bacterial ability to produce biofilms that act as a physical barrier to increase bacterial tolerance to these molecules protecting bacteria from death [21, 24, 26]. For *S. aureus* for example, a sub-inhibitory concentration of β-lactams increased 10 times the production of biofilm [22], and amoxicillin increased bacterial biofilm and altered biofilm composition [28].

Here we showed that *M. tomentosa* EtOAc fraction also stimulated biofilm production by two strains of *S. aureus*, showing that this partition generated a stressful environment for bacteria. Besides our work, it was demonstrated that *Microdesmia rigida* leaf extract (2048 μg/mL) was able to modulate biofilm production by different species of *Candida* [18]. From the nine tested *Candida* spp., three exhibited an increase of biofilm production, while in three others the biofilm production was decreased. Interestingly, this extract also contained glycosylated flavons myricetin-3-*O*-rhamnoside (myricitrin) and myricetin-*O*-hexoside (Table S1), and myricitrin was the most abundant molecule of the extract [18]. It remains unclear which mechanisms are related to the ability of this extract to induce or decrease biofilm production by bacteria and yeast, and if myricetin glycosylation contributes to the molecule activity.

Several studies have shown that myricetin has diverse biological activities such as anticarcinogen, antimutagen, antioxidant, anti-inflammatory, and it is also able to reduce platelet aggregation, viral infection and replication (revised by [1, 35]).

Hou et al. [19] have shown that free myricetin reduced LPS-induced pro-inflammatory molecules such as TNF-α, IL-6, IL-1β, COX-2 and iNOS (NOS2) by RAW264.7 macrophages in vivo, and also lung inflammation induced by LPS in a mice model. This effect was associated to the suppression of NF-κB p65 and AKT in NF-κB pathway and JNK, p-ERK and p38, in MAPK signaling pathway.

An anti-inflammatory activity was also observed for the glycosylated myricetin, myricitrin present in the leaf extract from *Myrica rubra*. The authors showed that the myricitrin enriched extract was able to inhibit TNF-α production by RAW 264.7 cells and reduce IgE levels in DO11.10 mice model [36]. Myricitrin was also able to reduce NO production and iNOS (NOS2) expression induced by LPS [11].

Similarly, we showed that *M. tomentosa*-enriched myricetin 3-*O*-xylosyl-rhamnoside fraction was able to reduce IL-6, TNF-α and NO production by RAW 264.7 macrophages stimulated with LPS, showing that both free and glycosylated myricetin have a potential anti-inflammatory effect. The suppression mechanisms underlying the anti-inflammatory effect of *M. tomentosa* EtOAc fraction are still under investigation.

Other studies have highlighted the importance of glycosylation for solubility, stability and biological activity of myricertin [41]. For example, [30], showed that myricetin 3-rhamnoside and myricetin 3-(6-rhamnosylgalactoside) are more effective than free myricetin to reduce HIV infection, since glycosylation may enhance flavonoid internalization by the cells allowing myricetin to act intracellularly.

Thus, isolation, structural and pharmacological studies of different glycosylated myricetin from plant sources is crucial to better understand the biological effects of these flavonoids and to improve the efficacy of these molecules for pharmacological purposes.

Collectively, our results indicate that the EtOAc fraction isolated from *M. tomentosa* leaves could act reducing bacterial survival and also inhibiting inflammatory response in vitro. Thus, we demonstrate that *M. tomentosa* leaf extract presents potential pharmacological properties. Whether this activity is relevant in vivo, is a subject to be further investigated using animal models of experimental digestive infection.

## Conclusion

Our study showed that *M. tomentosa* flavonoid-rich leaves fraction inhibited bacterial cell growth and modulated bacterial biofilm production. Also, the fraction was able to reduce macrophage activation induced by LPS, suggesting an anti-inflammatory effect.

## Supplementary Information

**Additional file 1: Figure S1.** TLC profile of EtOAc partition from M. tomentosa leaves revealed with NP-PEG, using the elution system, ethyl acetate: acetone: water (25:8:2) (A) and pure ethyl acetate (B).

**Additional file 2: Figure S2.** Direct infusion electrospray ionization (ESI) on negative mode of M. tomentosa ethyl acetate fraction. (A) Full-spectrum; (B) MS2 of the ion m/z 595; (C) MS3 of the ion m/z 316.

**Additional file 3: Table S1. **Myricetin flavons containing *Licania* species.

## Data Availability

All data and material are available from the corresponding author to share upon request.

## Abbreviations

EtOAc: Ethyl acetate
BuOH: N-butanol
DMSO: Dimethyl sulphoxide
PBS: Phosphate buffer saline
TSA: Trypticase soy agar
MTT: (3-[4,5-Dimethylthiazol-2-yl]-2,5 diphenyl tetrazolium bromide)
DMEM: Dulbecco's Modified Eagle Medium
DI-ESI-IT-MS: Direct infusion mass spectrometry
MS: Mass spectrometry
ESI: ElectroSpray Ionization
TLC: Thin layer chromatography
TNF-α: Tumor nuclear factor alpha
IL-6: Interleukin six
IL-1β: Interleukin one beta
COX-2: Cyclooxygenase two
iNOS: Induced nitric oxide synthese
NO: Nitric oxide
NF-κB: Nucelar factor κB
AKT: Protein kynase b
JNK: C-Jun N-terminal cinase
MAPK: Mitogen-activated protein kinase
