# Supplementary material for: Bactericidal and anti-inflammatory effects of Moquilea tomentosa Benth. flavonoid-rich leaf extract
Source: BMC Complement Med Ther. 2023 May 10;23:153. doi: 10.1186/s12906-023-03968-z (PMC10173630; doi:10.1186/s12906-023-03968-z)
Supplement: Supplementary file 1 — Additional file 1: Figure S1. TLC profile of EtOAc partition from M. tomentosa leaves revealed with NP-PEG, using the elution system, ethyl acetate: acetone: water (25:8:2) (A) and pure ethyl acetate (B). [file 12906_2023_3968_MOESM1_ESM.doc]

**Figure S1.** TLC profile of EtOAc partition from *M. tomentosa* leaves revealed with NP-PEG, using the elution system, ethyl acetate: acetone: water (25:8:2) (A) and pure ethyl acetate (B)


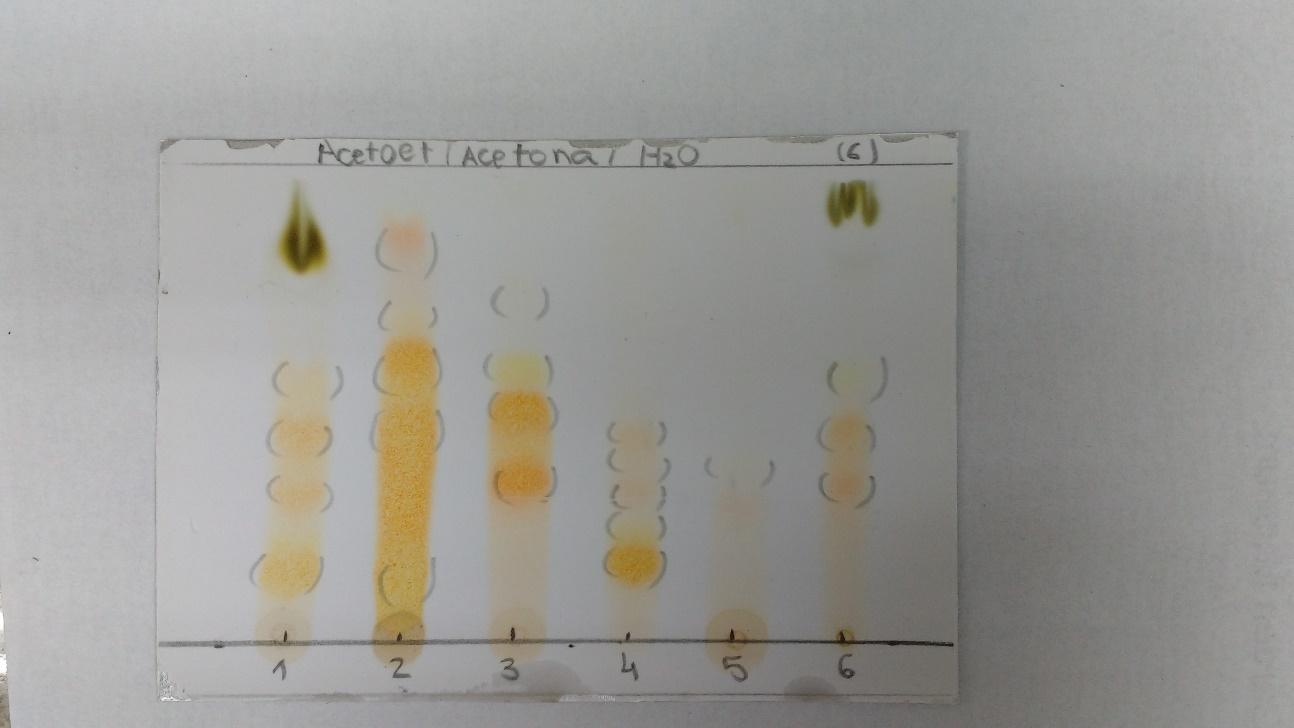

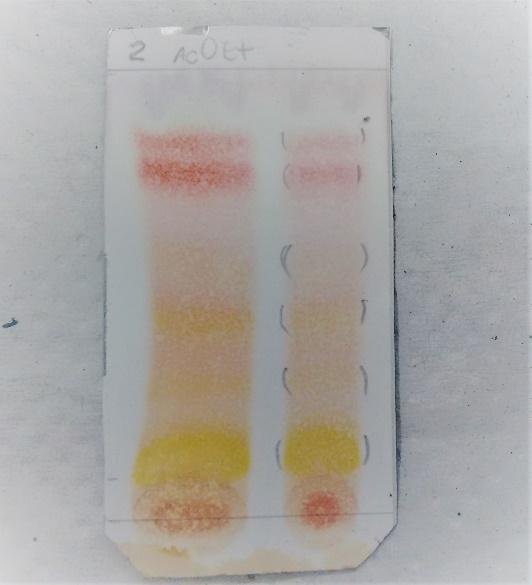


A

B
