# Supplementary material for: Bactericidal and anti-inflammatory effects of Moquilea tomentosa Benth. flavonoid-rich leaf extract
Source: BMC Complement Med Ther. 2023 May 10;23:153. doi: 10.1186/s12906-023-03968-z (PMC10173630; doi:10.1186/s12906-023-03968-z)

Figure S2. Direct infusion electrospray ionization (ESI) on negative mode of *M. tomentosa* ethyl acetate fraction. (A) Full-spectrum; (B) MS^2 of the ion *m/z* 595; (C) MS^3 of the ion *m/z* 316.

A

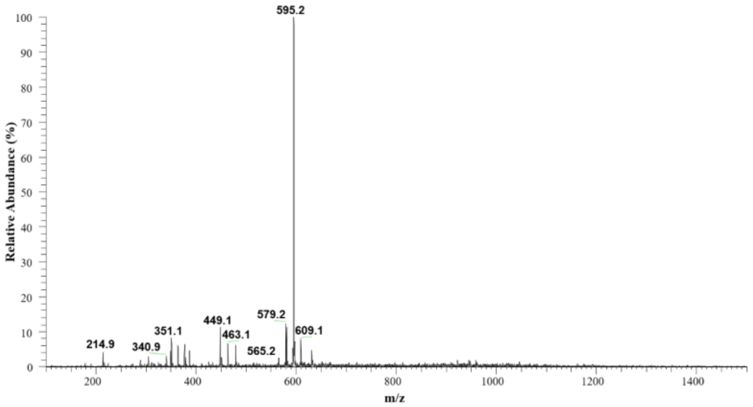

B

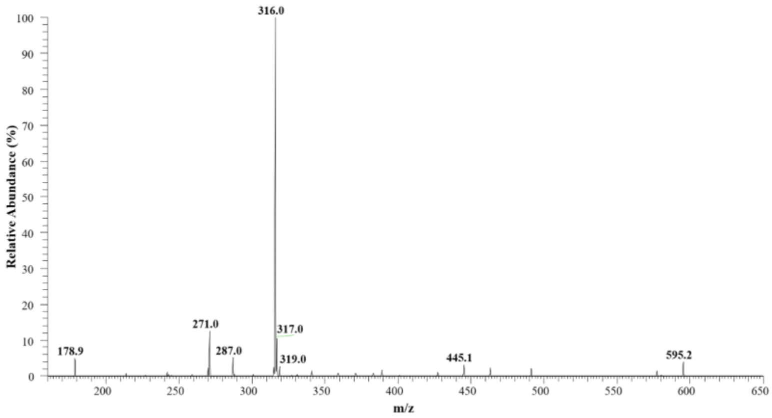

C

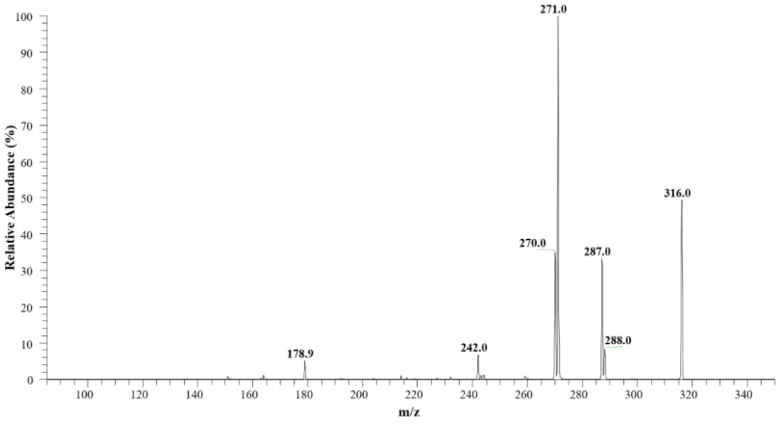

Supplement: Supplementary file 2 — Additional file 2: Figure S2. Direct infusion electrospray ionization (ESI) on negative mode of M. tomentosa ethyl acetate fraction. (A) Full-spectrum; (B) MS2 of the ion m/z 595; (C) MS3 of the ion m/z 316. [file 12906_2023_3968_MOESM2_ESM.pdf]
