# Supplementary material for: Bactericidal and anti-inflammatory effects of Moquilea tomentosa Benth. flavonoid-rich leaf extract
Source: BMC Complement Med Ther. 2023 May 10;23:153. doi: 10.1186/s12906-023-03968-z (PMC10173630; doi:10.1186/s12906-023-03968-z)
Supplement: Supplementary file 3 — Additional file 3: Table S1. Myricetin flavons containing Licania species. [file 12906_2023_3968_MOESM3_ESM.doc]

**Supplement material**

**Table S1.** Myricetin flavons containing *Licania* species.

| **Licania species and updated names** | **Identified flavon** | **Molecular weight* (g/mol)** | **Reference** |
| --- | --- | --- | --- |
| ***Leptobalanus apetalus* (= *Licania apetala*)** | Myricetin-4’-O-α-rhamnoside | 464 | Braca et al., 2002 |
| ***L. carii*** | Myricetin-3-glucoside | 480 | Bilia *et al.*, 1996**^a^** |
|  | Myricetin-3-galactoside | 480 |  |
|  | Myricetin-3-(2”-xylosyl) rhamnoside | 596 |  |
|  | Myricetin-3-rutinoside | 626 |  |
|  | Myricetin-3’-methyl-3-rutinoside | 642 |  |
| ***L. densiflora*** | 3’,4’-dimethylmyricetin-3-*O*-β-D-glucopyranoside | 502 | Braca *et al.*, 1999^a^  Braca *et al.*, 2001 |
|  | Myricetin 3-*O*-α -L-(2-*O*-α-L-rhamnopyranosyl)-rhamnopyranoside | 600 |  |
|  | Myricetin 3’,5’-dimethylether-3-*O*-rhamnoside | 478 |  |
|  | Myricetin 4’-methylether-3-*O*-rhamnoside | 478 |  |
|  | Myricetin 3’-methylether-3-*O*-glucoside | 480 |  |
|  | Myricetin 3’-methylether-3-*O*-galactoside | 480 |  |
|  | Myricetin 3’,5’-dimethyl ether-3-*O*-glucoside | 494 |  |
| ***Hymenopus heteromorphus***  **(= *L. heteromorpha*)** | Myricetin-3-rhamnoside | 464 | Braca *et al.*,1999^b^  Braca et al.,1999^c^ |
|  | Myricetin-3-galactoside | 480 |  |
|  | Myricetin-4’-methoxy-3-galactoside | 480 |  |
|  | Myricetin-4’-methoxy-3-glucoside | 480 |  |
|  | Myricetin-4’-methoxy-3-rhamnoside | 464 |  |
|  | Myricetin-3,4’-di-*O*-α-L-rhamnoside | 612 |  |
|  | Myricetin 7-methylether 3,4'-di-*O*-α-L-rhamnopyranoside | 616 |  |
|  | Myricetin 3,4'-di-*O*-α-L-  rhamnopyranoside | 610 |  |
|  | Myricetin 4'-methylether-3-O-β-D-  galactopyranoside | 494 |  |
| ***L licaneaeflora*** | Myricetin-3-*O*-α-arabinoside | 450 | Braca et al., 2002 |
|  | Dihydromyricetin-3-*O*-rhamnoside | 466 |  |
|  | Myricetin-3-*O*-β-galactoside | 480 |  |
| ***Microdesmia rigida***  **(= *L. rigida*)** | Myricetin-3-O-rhamnoside | 463 | Freitas et al., 2019 |
|  | Myricetin-*O*-hexoside | 479 |  |
| ***Moquilea tomentosa***  **(= *L. tomentosa*)** | Myricetin 3-*O*-xylosyl-rhamnoside | 596 | This study |
| ***Moquilea pyrifolia***  **(= *L. pyrifolia*)** | Myricetin | 318 | Bilia *et al*., 1996**^b^** |
|  | Myricetin-3-rhamnoside | 464 |  |
|  | Myricetin-3-(2’-xylosyl)-rhamnoside | 596 |  |

*Molecular weight values according to PubChem database or references.
